# Healthcare Workers’ Perceptions of the Effectiveness of Personal Protective Equipment in Reducing the Risk of COVID-19 Infection from 2020 to 2022

**DOI:** 10.3390/ijerph23060737

**Published:** 2026-05-31

**Authors:** Ndabereye Aubin Ndizeye, Makhutsisa Charlotte Mokoatle

**Affiliations:** Department of Environmental Health, Faculty of Health Sciences, University of Johannesburg, Johannesburg 2000, South Africa; charlottem@uj.ac.za

**Keywords:** healthcare workers (health personnel), personal protective equipment, COVID-19

## Abstract

**Highlights:**

**Public health relevance—How does this work relate to a public health issue?**

- Healthcare workers (HCWs) are at considerable risk of occupational exposure to various pathogens because of insufficient Personal Protective Equipment (PPE) and increased infection risks. They face difficulties in acquiring enough PPE that meets quality and quantity standards. Due to limited supplies, they often reuse PPE, increasing their risk of contracting Coronavirus Disease of 2019 (COVID-19).
- HCWs are experiencing confusion and declining trust because of frequent updates to patient treatment guidelines and inconsistent PPE instructions. This undermines their confidence in proper PPE use, possibly causing them to improvise and jeopardize their protection. Such practices also negatively influence how they perceive PPE’s overall effectiveness.

**Public health significance—Why is this work of significance to public health?**

- In the broader context of public health, protecting healthcare workers is essential for maintaining public trust and the efficiency of the health system during pandemics, as well as ensuring their safety. Lack of confidence in PPE can lead to burnout, increased absenteeism, and even staff attrition, all of which strain healthcare delivery. Additionally, the study’s findings on perception differences among racial and occupational groups suggest that risk communication strategies may not be effectively reaching all HCWs, underscoring the need for inclusive and context-aware approaches. Therefore, addressing perceptions is a structural and policy priority for pandemic preparedness and response, alongside overcoming behavioral barriers.
- The study’s findings highlight critical public health concerns about healthcare systems’ preparedness to protect frontline workers during infectious disease outbreaks. Despite a statistically significant correlation between positive perceptions and lower infection rates, only 57.07% of HCWs believed that PPE was effective in preventing COVID-19 infection. This indicates a lack of institutional support, risk communication, and training. These issues have serious implications for occupational health, as healthcare workers who do not see PPE as effective are more likely to ignore proper protective measures, increasing their risk of infection and the spread of nosocomial infections.

**Public health implications—What are the key implications or messages for practitioners, policy makers, and/or researchers in public health?**

- Healthcare facilities must focus on ongoing, practical PPE training for all healthcare workers, including support and secretarial staff who might often be overlooked. This training should highlight the benefits of PPE, address perceived barriers with problem-solving and clear demonstrations, and boost awareness of infection risks and severity.
- In resource-limited settings, healthcare facilities must ensure a consistent and equitable supply of PPE. This involves establishing monitoring systems to track PPE availability at the ward level, decentralizing PPE distribution, and implementing emergency procurement procedures. Daily use and supply of PPE by HCWs, especially face masks, help lower the risk of COVID-19 infection.

**Abstract:**

**Background/Objectives:** Healthcare workers (HCWs) face occupational hazards that increase their risk of Coronavirus Disease of 2019 (COVID-19). This study aims to evaluate HCWs’ perceptions of the effectiveness of Personal Protective Equipment (PPE) in preventing COVID-19 infection and to identify risk factors associated with HCW infection. **Methods:** A cross-sectional study design was used, with a structured, self-administered, closed-ended questionnaire to collect retrospective data for the period 2020 to 2022 at a tertiary hospital in Johannesburg, South Africa. **Results:** PPE was effective in reducing COVID-19 infection, according to 230 (57.07%) participants, while 173 (42.93%) disagreed. A significant association (*p* = 0.034) with a small effect size (Cramer’s V = 0.161) was found between the number of HCWs infected with COVID-19 and their perceptions of PPE’s effectiveness. White HCWs were more likely to perceive PPE as effective than Black HCWs (AOR = 3.82, *p* = 0.046). Support and clerical staff reported higher perceived effectiveness of PPE (AOR = 2.98, *p* = 0.040). **Conclusions:** HCWs encountered COVID-19 infections and various challenges that necessitate interventions and policies to safeguard them in hospital settings and ensure prompt virus management, including ensuring sufficient PPE supplies. The perceptions of PPE effectiveness among HCWs are shaped by an interplay of institutional practices, personal beliefs, and structural factors. These perceptions are closely tied to essential elements such as training, reliable PPE availability, and regular hand hygiene practices, underscoring the need to address both systemic and behavioral dimensions.

## 1. Introduction

Protecting healthcare workers (HCWs) from exposure to Severe Acute Respiratory Syndrome Coronavirus 2 (SARS-CoV-2) while caring for patients is crucial for managing Coronavirus Disease of 2019 (COVID-19). However, hospitals faced challenges securing adequate Personal Protective Equipment (PPE) and implementing infection prevention and control (IPC) guidelines. COVID-19 infections among HCWs continued to be reported, placing a significant strain on health systems in many countries [1]. In May 2020, approximately 152,888 HCWs were reported infected worldwide, and by November 2020, nearly 300,000 HCWs had been infected globally [2]. Global data on the exact number of HCWs infected with COVID-19 since the pandemic began are difficult to determine due to inconsistent reporting, underreporting, and differences in testing across countries. A review covering data from January 2021 through 2024 estimated that about 11% to 14% of HCWs worldwide were infected with SARS-CoV-2, with infection rates varying widely by region and variant, reaching up to 59% in some studies [3]. Many countries stopped providing regular updates on HCW-specific infections later in the pandemic, making it difficult to provide a single, definitive total by 2026. Despite the growing body of evidence on COVID-19 infections among HCWs globally, there is limited context-specific evidence on how HCWs perceive the effectiveness of PPE and how these perceptions influence their risk of infection, particularly in South African hospital settings, such as Gauteng Province. Furthermore, few studies simultaneously examine both behavioral (perceptions) and occupational risk factors within a theoretical framework, limiting the ability to design targeted interventions.

The Health Belief Model (HBM) is a widely used theoretical framework for understanding health-related behaviors. It holds that individuals’ engagement in preventive actions is shaped by perceived susceptibility to a disease, perceived severity of its consequences, perceived benefits of preventive measures, and perceived barriers to their implementation. In this study, the HBM served as the conceptual framework to examine how HCWs’ perceptions—specifically perceived susceptibility to COVID-19, perceived severity of the disease, perceived benefits of PPE, and perceived barriers to its use—may influence adherence to protective measures and, ultimately, the risk of infection.

Occupational factors such as insufficient PPE, improper PPE use, work overload, inadequate training, and poor infection control contributed to the high infection rates among HCWs [4]. In Africa, over 10,000 HCWs contracted COVID-19, with Niger (4.2%), Chad (5%), Liberia (5%), Algeria (5.3%), Tanzania (11%), and South Africa (23.4%) reporting the highest proportions of HCW infections [5]. In South Africa, COVID-19 cases among HCWs rose threefold compared to other African nations, with a rate exceeding 511 per 10,000 cases as of 2020 [2]. Most studies from South Africa are from various provinces, but few focus specifically on Gauteng. For example, a survey in the Eastern Cape found that 34.6% of HCWs had Polymerase Chain Reaction (PCR) confirmed COVID-19 [6]. Another report indicated that HCWs represented 3.8% (6363) of hospitalized COVID-19 patients across several hospitals [7]. Furthermore, a study by Reese et al. across five districts in South Africa found that 16% of HCWs contracted COVID-19 [8]. Despite these findings, there remains limited evidence on the determinants of PPE use and perceptions of its effectiveness among HCWs in Gauteng Province, particularly within large tertiary hospitals such as Chris Hani Baragwanath Academic Hospital (CHBAH).

Poor PPE use in the healthcare system can lead to COVID-19 infections [9]. In South Africa, some facilities struggled to provide sufficient PPE due to supply chain issues. HCWs in certain hospitals also lack training in proper PPE use [10]. Other factors associated with poor PPE use include age, comorbidities, race, sex, and perceptions of PPE. A 2016 study found that proper PPE use significantly reduces the risk of hospital infection by 85%. However, challenges such as resource shortages, improper use, discomfort, and compliance issues persist, especially in low- and middle-income countries (LMICs) [10]. Additionally, a Saudi Arabian study [11] showed that while HCWs generally believe PPE is effective, their confidence is often affected by concerns about its quality and availability, particularly during periods of high transmission. Access to infection-control training and the frequency of PPE use are strongly linked to perceptions of PPE. This highlights the importance of ongoing education and administrative support in promoting positive attitudes and proper PPE use. To better understand HCWs’ perceptions of PPE effectiveness, policies, and strategies, and of CHBAH’s efforts to ensure HCW safety, our study was conducted across various departments, roles, and professions. Therefore, this study aimed to assess HCWs’ perceptions of PPE’s effectiveness in reducing COVID-19 infection and to identify associated risk factors, using the HBM as a conceptual framework to better understand behavioral and occupational determinants of infection risk.

## 2. Materials and Methods

The study was conducted at one of the 40 tertiary hospitals in Johannesburg, Gauteng province, South Africa, namely CHBAH (also known as Baragwanath Hospital), retrospectively for the period between 2020 and 2022. The study involved HCWs who were permanently employed at the hospital, including nurses doctors, administrators, and allied staff such as laboratory technicians, radiographers, support staff, and clerical workers. Participants came from various departments, including maternity, obstetrics-gynecology, surgery, internal medicine, pediatrics, laboratory services, pharmacy, intensive care, neurology, transport, and administration. Inclusion criteria were HCWs permanently employed at CHBAH and available during the study period. Exclusion criteria included temporary staff, students, and those who declined participation. A convenient non-probability sampling method was used in this study, and all HCWs willing to participate completed the survey. We used Epi Info version 5.5.9, a statistical software, to calculate the sample size. With a population size of 6760 and an acceptable error margin of 5%, and 1 cluster, CHBAH, the estimated sample size at a 95% confidence level was 363. A 10% contingency was added, resulting in a final sample size of 399 HCWs. However, more HCWs consented, completed, and returned the questionnaire, yielding a total of 403 responses (over 100%), and all were analyzed. Data were collected using a structured, self-administered questionnaire divided into sections: socio-demographic characteristics (age, race, sex, occupational categories, comorbidities), perceptions of proper PPE use, and risk factors related to COVID-19 infection. Perception of PPE effectiveness was defined as HCWs’ self-reported belief regarding the ability of PPE (mask, gloves, gown, face shield) to prevent COVID-19 transmission during patient care. This construct was operationalized using Likert-scale items assessing perceived effectiveness and confidence in PPE use. Responses to perception-related items were coded on a 4-point Likert scale (1 = rarely to 4 = always as recommended). A composite perception score was calculated by summing the responses across relevant items. The total score was then categorized into high and low perception levels based on the median value. It was designed to take no more than 30 min for HCWs to complete. A letter was sent to the hospital requesting permission to conduct the study, addressed to the Director of the Gauteng Department of Health. After obtaining approval from the hospital’s Chief Executive Officer, the researcher visited each department to introduce the study. The study was briefly explained to HCWs using information sheets, and consent forms were distributed to confirm their agreement to participate. Hard copies of the questionnaires were provided for HCWs to complete at their convenience and deposit in a designated collection box. The questionnaire was designed and adapted from similar tools developed by the World Health Organization (WHO) and the Centre for Disease Control (CDC) [12]. It was developed based on previously validated instruments from the WHO and CDC guidelines and adapted to the local context. It comprised four main domains: (1) socio-demographic characteristics, (2) occupational exposure and infection history, (3) perceptions of PPE effectiveness based on HBM constructs, and (4) adherence to infection prevention and control practices. The researcher periodically visited the hospital to check for completed questionnaires. Participants who agreed to take part signed a consent form, and the form was collected daily until the desired sample size was reached. Participants were given one month to complete the survey. Measures were taken to minimize information bias, including anonymous data collection and standardized instructions provided to participants.

Data from the questionnaires were collected and initially coded in Microsoft Excel, then exported to the Statistical Package for the Social Sciences (SPSS, version 29) for analysis. A statistician independently reviewed the analytical procedures and results to ensure accuracy and consistency. Responses to open-ended questions were analyzed using content analysis to identify recurring themes.

Descriptive statistics, including frequencies and proportions, were used to summarize participants’ characteristics and perceptions of PPE effectiveness. The internal consistency of the survey instrument was assessed using Cronbach’s alpha, yielding a coefficient of 0.90, indicating excellent reliability and exceeding the commonly accepted threshold of 0.70 [13]. A pilot study was conducted with 25 participants who were not included in the final sample to evaluate the questionnaire’s clarity, consistency, and applicability. Content validity was ensured through expert review by clinicians and epidemiologists, while construct validity was supported by alignment of questionnaire items with the HBM framework.

To identify factors associated with HCWs’ perception of PPE effectiveness in reducing COVID-19 infection, both bivariate and multivariable logistic regression analyses were performed. Crude odds ratios (COR) and adjusted odds ratios (AOR), along with their 95% confidence intervals (CI), were reported as measures of association. Variables with a *p*-value < 0.25 in the bivariate analysis were included in the multivariable model to avoid prematurely excluding potentially important predictors and to ensure adequate control for confounding.

Multicollinearity among independent variables was assessed using variance inflation factors (VIFs), and no evidence of problematic multicollinearity was observed (all VIFs < 5). Model fit was evaluated using standard goodness-of-fit measures, including the Hosmer–Lemeshow test. A *p*-value < 0.05 was considered statistically significant at the 95% confidence level.

Given the cross-sectional design of the study, the reported associations should be interpreted with caution and do not imply causality.

## 3. Results

### 3.1. Sociodemographic and Occupational Characteristics

A total of 403 HCWs were included in the study. Most participants were female (70.5%), with a mean age of 40.0 ± 8.1 years. The largest age group was 31–39 years (43.4%), and most were single (61.0%). The study population was predominantly Black (87.6%).

Regarding occupational characteristics, nurses constituted the largest professional group (45.9%), followed by medical doctors (20.8%). More than half of participants (55.3%) began employment at CHBAH before the COVID-19 pandemic, with work experience distributed relatively evenly across all categories. The overwhelming majority of participants (92.1%) reported having undergone COVID-19 testing between 2020 and 2022.

Table 1 presents the distribution of sociodemographic and occupational characteristics by perceived PPE effectiveness. No statistically significant differences were observed between the two groups across most variables, including gender (*p* = 0.303), age (*p* = 0.654), marital status (*p* = 0.248), race (*p* = 0.433), work experience (*p* = 0.383), occupational category (*p* = 0.125), or COVID-19 testing history (*p* = 0.784). However, a statistically significant association was found with the time of employment commencement at CHBAH: a higher proportion of those who started before COVID-19 reported PPE as ineffective compared to those who started during the pandemic (61.3% vs. 38.7%; *p* = 0.038) (Table 1).

### 3.2. Exposure and Infection Prevention Characteristics

Table 2 presents exposure, infection prevention practices, and PPE-related factors, stratified by perceived PPE effectiveness. Many participants reported contact with a COVID-19 patient (96.3%) and PPE use during that contact (95.5%). Overall, 60.3% of participants reported receiving occupational health training (OHT), and 63.5% had received PPE-specific training.

Significant differences were observed between participants who perceived PPE as effective and those who perceived it as ineffective across several key variables. Those who perceived PPE as effective were more likely to have received OHT (67.0% vs. 51.5%; *p* = 0.002) and PPE-specific training (77.4% vs. 45.1%; *p* < 0.001). Hand hygiene practices also differed significantly between groups: a higher proportion of participants who perceived PPE as effective reported always practicing hand hygiene (83.9% vs. 69.4%), whereas fewer participants in the other group reported rarely practicing hand hygiene (0.4% vs. 8.1%; *p* < 0.001). Access to a daily PPE supply was substantially higher among those who perceived PPE as effective (89.1% vs. 63.0%; *p* < 0.001).

No statistically significant differences were observed in contact with COVID-19 patients (*p* = 0.766), PPE use during contact (*p* = 0.894), or prior PPE experience before the pandemic (*p* = 0.072). These findings suggest that structured training and reliable access to resources are key determinants of healthcare workers’ positive perceptions of PPE effectiveness.

### 3.3. Correlation Between PPE Usage and COVID-19 Infection

The association between HCWs’ perception of PPE effectiveness and the number of self-reported COVID-19 infections was statistically significant (*p* = 0.034), but the association was weak (Cramér’s V = 0.161).

Among HCWs who did not perceive PPE as effective, 53.76% (n = 93) reported never being infected, compared with 59.13% (n = 136) among those who perceived PPE as effective. Differences were also observed across infection-frequency categories, though these were modest (Table 3).

### 3.4. Bivariate Analysis

The bivariate analysis examined factors associated with healthcare workers’ perceptions of PPE effectiveness (Table 4). Access to occupational health services (OHS) during COVID-19 was associated with higher odds of perceiving PPE as effective (COR = 1.91, 95% CI: 1.28–2.87, *p* = 0.002). Similarly, receipt of adequate PPE training (COR = 4.17, 95% CI: 2.71–6.41, *p* < 0.001) and daily hospital-provided PPE (COR = 4.81, 95% CI: 2.87–8.08, *p* < 0.001) were associated with higher perceived effectiveness.

In contrast, HCWs who reported washing or sanitizing their hands “most of the time” (COR = 0.54, 95% CI: 0.32–0.91, *p* = 0.019) or “rarely” (COR = 0.04, 95% CI: 0.01–0.34, *p* = 0.003) had lower odds of perceiving PPE as effective than those who consistently adhered to recommended hand hygiene practices.

Other variables, including prior PPE use before COVID-19, contact with COVID-19-positive patients, and PPE use during such contact, were not statistically associated with perceived PPE effectiveness. Variables with *p*-values < 0.25 were considered for inclusion in the multivariable model (Table 4).

### 3.5. Multivariate Analysis

The multivariable analysis identified factors independently associated with healthcare workers’ perceptions of PPE effectiveness. White HCWs had higher odds of perceiving PPE as effective than Black HCWs (AOR = 3.82, 95% CI: 1.02–14.24, *p* = 0.046). However, the wide confidence interval suggests limited precision of this estimate.

Support and clerical staff also had higher odds of perceiving PPE as effective (AOR = 2.98, 95% CI: 1.05–8.44, *p* = 0.040). Receipt of adequate PPE training remained strongly associated with higher perceived effectiveness (AOR = 3.40, 95% CI: 1.99–5.80, *p* < 0.001), as did daily PPE supply (AOR = 3.48, 95% CI: 1.87–6.46, *p* < 0.001).

In contrast, HCWs who reported rarely practicing hand hygiene had lower odds of perceiving PPE as effective (AOR = 0.11, 95% CI: 0.01–0.97, *p* = 0.047). However, this estimate should be interpreted cautiously, given the wide confidence interval.

Other variables, including gender, marital status, work experience, occupational health training during COVID-19, and prior PPE experience, were not independently associated with perceived PPE effectiveness (Table 4).

## 4. Discussion

The findings of this study show that most HCWs (57.07%) believe that PPE effectively reduces the risk of contracting COVID-19. This research highlights important knowledge, structural, and behavioral gaps in IPC and reveals conflicting opinions. Despite the global focus during the pandemic and most HCWs reporting this level of perceived effectiveness, this level is somewhat lower than expected, even though PPE is generally recognized as a key element in preventing occupational exposure to infectious diseases. This aligns with other research indicating that PPE alone is insufficient to ensure healthcare providers’ confidence and compliance unless combined with proper working conditions, adequate training, and trust in healthcare systems [14,15]. Similar findings have been reported in African settings. For instance, a cross-sectional study conducted in the Democratic Republic of the Congo found that although HCWs had high levels of knowledge about COVID-19, only 54.9% reported consistent use of PPE, highlighting a gap between knowledge and actual preventive practices [16]. Furthermore, the significant association between PPE perception and infection status is consistent with earlier pandemic-related studies. In this research, HCWs who believed in the effectiveness of PPE were significantly less likely to report COVID-19 infections. This suggests that a positive perception may be associated with more consistent and correct PPE use, although no causal relationship can be established due to the cross-sectional design. Similar findings were reported by Chou et al. [17], who noted that HCWs with strong beliefs in institutional IPC protocols and proper PPE practices had notably lower infection rates. This shows an association between HCWs’ perception and infection history. However, this association should be interpreted with caution, as the temporal relationship between perception and infection cannot be established. Additionally, a meta-analysis by Gomez-Ochoa et al. [18] reinforced this conclusion, reporting that the odds of contracting COVID-19 were significantly lower among HCWs who strictly adhered to PPE protocols than among those who did not.

These findings are consistent with evidence from cross-sectional studies conducted during the COVID-19 pandemic, which have similarly reported associations between risk perception, adherence to preventive measures, and infection outcomes, while emphasizing the methodological limitations inherent to observational designs.

In the bivariate analysis, occupational roles appeared to influence perceptions of PPE. Medical doctors and laboratory workers were significantly more likely to view PPE as effective. This higher perception among doctors and laboratory workers is probably due to more frequent infection-control training and a better understanding of disease transmission mechanisms. A study also reported that physicians (87%) had a good level of knowledge about COVID-19 and demonstrated better compliance with PPE protocols, possibly because of their clinical background and ongoing medical education [19]. Conversely, laboratory workers’ high perception could stem from their frequent use of PPE during work, given their environment and the nature of their tasks, which leads to a stronger reliance on PPE for self-protection. However, these factors were no longer significant in the multivariate analysis as predictors of effective PPE use among HCWs.

One of the most important findings was the essential role of institutional support, especially through adequate PPE training (AOR = 3.40, 95% CI: 1.99–5.80, *p* < 0.001) and daily PPE supplies (AOR = 3.48, 95% CI: 1.87–6.46, *p* < 0.001). These factors were among the main predictors of perceiving PPE as effective in reducing COVID-19. HCWs who received thorough PPE training and had daily PPE supplies were much more likely to view PPE as effective. These findings suggest that strengthening PPE training programs and ensuring consistent supply may be important considerations for improving HCWs’ perceptions and adherence to protective measures. Therefore, these findings show that perceived PPE effectiveness, which relies on proper use and compliance, significantly improves when HCWs feel supported through training and access to equipment. This aligns with a study that emphasizes the importance of PPE training quality and frequency for proper use and confidence [20]. Additionally, the idea that when HCWs trust PPE availability and quality, their risk perception decreases, leading to greater psychological readiness and adherence to safety guidelines, remains valid [14].

Differences in perceptions of PPE effectiveness across racial groups were observed in this study. White HCWs were more likely than their Black counterparts to view PPE as effective (AOR = 3.82, 95% CI: 1.02–14.24, *p* = 0.046). These differences should be interpreted cautiously, as the study was not designed to explore underlying structural or socioeconomic determinants, and residual confounding cannot be ruled out. In addition, self-reported measures may have influenced these findings through recall or social desirability bias. These findings are consistent with previous studies reporting disparities in occupational safety perceptions [21]; however, caution is needed when interpreting these differences, as the study did not directly assess underlying social or structural determinants. Addressing these disparities may be important for promoting equitable adoption of protective measures across healthcare groups.

Another key behavioral component identified in the study is hand hygiene. Those who followed recommended hand hygiene practices were less likely to report low perceived effectiveness of PPE (AOR = 0.11, 95% CI: 0.01–0.97, *p* = 0.047), suggesting an association between adherence to infection-prevention behaviors and more favorable perceptions of PPE. This may reflect behavioral clustering in IPC, in which engagement in one protective behavior is associated with engagement in other protective behaviors. This pattern aligns with previous research showing a strong link between general infection-control behavior and risk perception [22]. It is also consistent with findings from other cross-sectional studies in low- and middle-income countries, where adequate knowledge does not always translate into appropriate preventive practices, particularly regarding PPE use [16].

Further research is needed to better understand the contextual and structural factors that may explain these differences. However, the magnitude of some associations observed in this study was modest, so findings should be interpreted with caution despite their statistical significance.

However, the perception of PPE effectiveness was not significantly influenced by gender, marital status, years of work experience, or having access to occupational health services during COVID-19. This contrasts with some earlier studies, which suggested that longer work experience and access to support services were associated with greater confidence in infection control measures [23].

## 5. Strengths

This study addresses a significant public health issue faced by frontline HCWs at CHBAH, one of Africa’s largest tertiary hospitals. To improve the statistical power and representativeness of the findings, the study used a sufficiently large sample size (n = 403), determined with validated epidemiological software (Epi Info). It ensures a broad range of perspectives and experiences by including a diverse sample of HCWs from various departments and roles. Content validity is further strengthened by using a self-administered questionnaire adapted from WHO and CDC resources, and the instrument’s clarity and reliability are confirmed through pilot testing.

## 6. Limitations

This study has limitations that should be considered when interpreting the findings. First, the cross-sectional design limits the ability to establish temporal relationships or infer causality between exposures (e.g., PPE use, training) and outcomes such as COVID-19 infection. In particular, the observed association between HCWs’ perception of PPE effectiveness and infection status was weak and should not be interpreted as clinically meaningful or as evidence of causation.

Second, the observed associations may be influenced by residual confounding, as not all potential confounders could be fully accounted for in the analysis. In addition, some statistically significant associations had relatively small effect sizes or wide confidence intervals, indicating limited precision and reducing their potential clinical or practical relevance.

Third, self-reported data may introduce recall and social desirability biases, as participants may overreport adherence to protective measures or underreport prior infection. Furthermore, convenience sampling, which included only healthcare workers who were available and agreed to participate during data collection, may have introduced selection bias and limited the generalizability of the findings.

Finally, the lack of objective verification of COVID-19 infection status (e.g., through medical records or serological testing) may have affected the accuracy of reported outcomes, particularly given resource constraints.

## 7. Conclusions

This study illustrates how the intricate interplay among institutional practices, personal beliefs, and structural factors shapes HCWs’ perceptions of PPE effectiveness. Although a slight majority considered PPE effective, this belief was strongly linked to proper training, adequate daily PPE supplies, and consistent hand hygiene, underscoring both systemic and behavioral influences. The HBM served as the framework for understanding how perceived benefits, barriers, and self-efficacy shape protective behaviors. The findings support behavioral theory-based interventions to enhance health outcomes, showing that a positive attitude correlates with reduced infection rates. The study also acknowledges limitations, including its cross-sectional design, which limits causal inference regarding PPE use, training, and COVID-19 outcomes. Self-reported data may lead to overreporting of compliance or underreporting of infections due to recall or social desirability biases. Including only HCWs willing and available to participate may introduce selection bias, limiting generalizability. Furthermore, due to budget constraints, the true infection rates could differ without verification from health records or serological tests. Despite these limitations, a key strength is addressing urgent public health concerns among HCWs at CHBAH, one of Africa’s largest tertiary hospitals. To improve statistical power and representativeness, the sample size was large (n = 403), calculated using validated epidemiological software (Epi Info). The study captures diverse viewpoints by including HCWs from various departments and roles. Content validity was ensured through a self-administered questionnaire adapted from WHO and CDC resources, and clarity and reliability were validated through pilot testing.

## Figures and Tables

**Table 1:** Sociodemographic and occupational characteristics of healthcare workers by perception of PPE effectiveness (N = 403).

Variable	Category	Total (N = 403), n (%)	PPE not Effective (n = 173), n (%)	PPE Effective (n = 230), n (%)	*p*-Value *
**Gender**	Female	284 (70.47)	128 (73.99)	156 (67.83)	0.303
	Male	118 (29.28)	45 (26.01)	73 (31.74)	
	Others	1 (0.25)	0 (0.00)	1 (0.43)	
**Age (years)**	23–30	45 (11.17)	16 (9.25)	29 (12.61)	0.654
	31–39	175 (43.42)	78 (45.09)	97 (42.17)	
	40–49	125 (31.02)	56 (32.37)	69 (30.00)	
	≥50	58 (14.39)	23 (13.29)	35 (15.22)	
**Marital status**	Married	157 (38.96)	73 (41.20)	84 (36.52)	0.248
	Single	246 (61.04)	100 (57.80)	146 (63.48)	
**Race**	Black	353 (87.59)	156 (90.17)	197 (85.65)	0.433
	Coloured	6 (1.49)	3 (1.73)	3 (1.30)	
	Indian	23 (5.71)	8 (4.62)	15 (6.52)	
	White	21 (5.21)	6 (3.47)	15 (6.52)	
**Work experience**	<5 years	144 (35.73)	57 (32.95)	87 (37.83)	0.383
	5–10 years	129 (32.01)	54 (31.21)	75 (32.61)	
	>10 years	130 (32.26)	62 (35.81)	68 (29.57)	
**Started working at CHBAH**	Before COVID-19	223 (55.33)	106 (61.27)	117 (50.87)	0.038
	During COVID-19	180 (44.67)	67 (38.73)	113 (49.13)	
**Occupational category**	Administrator	38 (9.43)	21 (12.14)	17 (7.39)	0.125
	Laboratory worker	18 (4.47)	5 (2.89)	13 (5.65)	
	Medical doctor	84 (20.84)	28 (16.18)	56 (24.35)	
	Nurse	185 (45.91)	83 (47.98)	102 (44.35)	
	Pharmacist	18 (4.47)	10 (5.78)	8 (3.48)	
	Physiotherapist	4 (0.99)	3 (1.73)	1 (0.43)	
	Radiographer	5 (1.24)	3 (1.73)	2 (0.87)	
	Support & clerical	45 (11.17)	19 (10.98)	26 (11.30)	
	Transport	6 (1.49)	1 (0.58)	5 (2.17)	
**Tested COVID-19 (2020–2022)**	No	32 (7.94)	13 (7.51)	19 (8.26)	0.784
	Yes	371 (92.06)	160 992.49)	211 (91.74)	

* *p*-values from chi-square test. PPE = Personal Protective Equipment; CHBAH = Chris Hani Baragwanath Academic Hospital.

**Table 2:** Exposure, infection prevention practices, and PPE-related factors among healthcare workers (N = 403).

Variable	Category	Total (N = 403), n (%)	PPE Not Effective (n = 173), n (%)	PPE Effective (n = 230), n (%)	*p*-Value *
**Occupational health training (OHT)**	No	160 (39.70)	84 (48.55)	76 (33.04)	0.002
	Yes	243 (60.30)	89 (51.45)	154 (66.96)	
**PPE training**	No	147 (36.48)	95 (54.91)	52 (22.61)	<0.001
	Yes	256 (63.52)	78 (45.09)	178 (77.39)	
**Contact with a COVID-19 patient**	No	15 (3.72)	7 (4.05)	8 (3.48)	0.766
	Yes	388 (96.28)	166 (95.95)	222 (96.52)	
**PPE use during contact**	No	18 (4.47)	8 (4.62)	10 (4.35)	0.894
	Yes	385 (95.53)	165 (95.38)	220 (95.65)	
**Hand hygiene**	Always	313 (77.67)	120 (69.36)	193 (83.91)	<0.001
	Most of the time	73 (18.11)	39 (22.54)	34 (14.78)	
	Occasionally	2 (0.50)	0 (0.00)	2 (0.87)	
	Rarely	15 (3.72)	14 (8.09)	1 (0.43)	
**Daily PPE supply**	No	89 (22.08)	64 (36.99)	25 (10.87)	<0.001
	Yes	314 (77.92)	109 (63.01)	205 (89.13)	
**PPE experience before COVID-19**	No	200 (49.75)	95 (54.91)	105 (45.85)	0.072
	Yes	202 (50.25)	78 (45.09)	124 (54.15)	

* *p*-values from chi-square test. PPE = Personal Protective Equipment; OHT = Occupational Health Training.

**Table 3:** Correlation between effectiveness of PPE Usage and COVID-19 infection (N = 403).

Times Infected with COVID-19	Effective Use of PPE		*p*-Value	Cramér’s (V)
	No (n = 173, 42.93%)	Yes (n = 230, 57.07%)		
0	93 (53.76)	136 (59.13)	0.034	0.161
1	49 (28.32)	74 (32.17)		
2	23 (13.29)	18 (7.83)		
3	7 (4.05)	1 (0.43)		
4	1 (0.58)	1 (0.58)		

**Table 4:** Factors associated with healthcare workers’ perception of PPE effectiveness in reducing COVID-19 infection (N = 403).

Factor	Category	COR (95% CI)	*p*-Value	AOR (95% CI)	*p*-Value
**Gender**	Female	1.00 (ref)	–	1.00 (ref)	–
	Male	1.33 (0.86–2.06)	0.202	1.11 (0.65–1.91)	0.705
**Age (years)**	23–30	1.00 (ref)	–	–	–
	31–39	0.69 (0.35–1.35)	0.277	–	–
	40–49	0.68 (0.34–1.38)	0.283	–	–
	≥50	0.84 (0.38–1.88)	0.671	–	–
**Marital status**	Married	1.00 (ref)	–	1.00 (ref)	–
	Single	1.27 (0.85–1.90)	0.250	1.14 (0.67–1.93)	0.631
**Race**	Black	1.00 (ref)	–	1.00 (ref)	–
	Coloured	0.79 (0.15–3.98)	0.777	0.52 (0.09–2.96)	0.460
	Indian	1.48 (0.61–3.59)	0.381	1.70 (0.52–5.52)	0.377
	White	1.98 (0.75–5.22)	0.168	3.82 (1.02–14.24)	0.046 *
**Started working at CHBAH**	Before COVID-19	1.00 (ref)	–	1.00 (ref)	–
	During COVID-19	1.53 (1.02–2.28)	0.038 *	1.02 (0.51–2.03)	0.961
**Work experience**	<5 years	1.00 (ref)	–	1.00 (ref)	–
	5–10 years	0.91 (0.56–1.48)	0.702	1.12 (0.55–2.27)	0.752
	>10 years	0.72 (0.44–1.16)	0.177	1.03 (0.46–2.31)	0.938
**Occupational category**	Administrator	1.00 (ref)	–	1.00 (ref)	–
	Laboratory worker	3.21 (0.95–10.81)	0.059	1.95 (0.39–10.81)	0.344
	Medical doctor	2.47 (1.13–5.41)	0.024 *	1.57 (0.63–3.91)	0.394
	Nurse	1.52 (0.75–3.06)	0.244	1.32 (0.75–3.06)	0.499
	Pharmacist	0.99 (0.32–3.05)	0.984	1.02 (0.32–3.05)	0.972
	Physiotherapist	0.41 (0.04–4.33)	0.460	0.14 (0.04–4.33)	0.171
	Radiographer	0.82 (0.12–5.51)	0.841	1.21 (0.12–5.51)	0.881
	Support & clerical	1.69 (0.71–4.04)	0.238	2.98 (1.05–8.44)	0.040 *
	Transport	6.18 (0.66–58.03)	0.111	3.00 (0.66–58.03)	0.357
**Tested COVID-19 (2020–2022)**	No	1.00 (ref)	–	–	–
	Yes	0.90 (0.43–1.88)	0.784	–	–
**Had OHT during COVID-19**	No	1.00 (ref)	–	1.00 (ref)	–
	Yes	1.91 (1.28–2.87)	0.002 *	1.07 (0.65–1.78)	0.786
**Received adequate PPE training**	No	1.00 (ref)	–	1.00 (ref)	–
	Yes	4.17 (2.71–6.41)	<0.001 *	3.40 (1.99–5.80)	<0.001 *
**Contact with a positive COVID-19 patient**	No	1.00 (ref)	–	–	–
	Yes	1.17 (0.42–3.29)	0.766	–	–
**If yes, were you using PPE**	No	1.00 (ref)	–	–	–
	Yes	1.07 (0.41–2.7)	0.894	–	–
**Wash or sanitize your hands**	Always	1.00 (ref)	–	1.00 (ref)	–
	Most of the time	0.54 (0.32–0.91)	0.019 *	0.58 (0.32–1.06)	0.075
	Rarely	0.04 (0.01–0.34)	0.003 *	0.11 (0.01–0.97)	0.047 *
**Daily PPE supply by the hospital**	No	1.00 (ref)	–	1.00 (ref)	–
	Yes	4.81 (2.87–8.08)	<0.001 *	3.48 (1.87–6.46)	<0.001 *
**Experience in** **using PPE before COVID-19**	No	1.00 (ref)	–	1.00 (ref)	–
	Yes	1.44 (0.97–2.14)	0.072	0.89 (0.54–1.45)	0.639

**Abbreviations:** COR = Crude Odds Ratio; AOR = Adjusted Odds Ratio; CI = Confidence Interval; Ref = Reference; PPE = personal protective equipment; OHT = Occupational Health Training; * Statistically significant at *p* < 0.05; CHBAH = Chris Hani Baragwanath Academic Hospital; COVID-19 = Coronavirus Disease of 2019.

## Data Availability

The original contributions made in this study are included in the article. For further questions, please contact the authors.

## Abbreviations

The following abbreviations are used in this manuscript:

**AOR**	Adjusted Odds Ratio
CEO	Chief Executive Officer
CHBAH	Chris Hani Baragwanath Academic Hospital
CI	Confidence Interval
COR	Crude Odds ratio
COVID-19	Coronavirus 2019
HCWs	Healthcare Workers
N	Number of Participants
*p*	Probability value
PPE	Personal Protective Equipment
WHO	World Health Organization
